# The relationship between vestibular function and topographical memory in older adults

**DOI:** 10.3389/fnint.2014.00046

**Published:** 2014-06-02

**Authors:** Fred H. Previc, Wesley W. Krueger, Ruth A. Ross, Michael A. Roman, Gregg Siegel

**Affiliations:** ^1^Biomedical Development CorporationSan Antonio, TX, USA; ^2^Ear Institute of TexasSan Antonio, TX, USA

**Keywords:** vestibular, topographical memory, hippocampus, Alzheimer disease, elderly

## Abstract

Research during the past two decades has demonstrated an important role of the vestibular system in topographical orientation and memory and the network of neural structures associated with them. Almost all of the supporting data have come from animal or human clinical studies, however. The purpose of the present study was to investigate the link between vestibular function and topographical memory in normal elderly humans. Twenty-five participants aged 70 to 85 years who scored from mildly impaired to normal on the Montreal Cognitive Assessment (MoCA) received three topographical memory tests: the Camden Topographical Recognition Memory Test (CTMRT), a computerized topographical mental rotation test (TMRT), and a virtual pond maze (VPM). They also received six vestibular or oculomotor tests: optokinetic nystagmus (OKN), visual pursuit (VP), actively generated vestibulo-ocular reflex (VOR), the sensory orientation test (SOT) for posture, and two measures of rotational memory (error in degrees, or RM°, and correct directional recognition, or RM→). The only significant bivariate correlations were among the three vestibular measures primarily assessing horizontal canal function (VOR, RM°, and RM→). A multiple regression analysis showed significant relationships between vestibular and demographic predictors and both the TMRT (*R* = 0.78) and VPM (*R* = 0.66) measures. The significant relationship between the vestibular and topographical memory measures supports the theory that vestibular loss may contribute to topographical memory impairment in the elderly.

## Introduction

The topographical orientation system, also known as the spatial navigation, topokinetic, and action-extrapersonal systems, is one of the four major networks in the brain governing our interaction with our 3D environment (Previc, 1998). It is believed to be comprised mainly of three posterior regions—the hippocampus, posterior cingulate, and parietal-temporal cortex (Berthoz, 1997; Previc, 1998)—and frontal-striatal structures such as the caudate nucleus (Maguire et al., 1998). It is the system that is responsible for scene and route memory, presence in the world, and topographical orientation in the plane of the Earth’s surface (Previc, 1998).

The most widely studied of the topographical regions is the hippocampus. Activation of the hippocampus occurs during recall of topographical routes (Maguire et al., 1997), and damage to it results in a profound amnesia for spatial landmarks, loss of spatial maps, and severe topographical disorientation (Aguirre and D’Esposito, 1999). In maintaining a cognitive map of the environment, the hippocampus integrates inputs from various sensory modalities, the two most important of these being distal visual inputs representing large regions of mainly the upper visual field (Previc, 1998; Arcaro et al., 2009) and those emanating from the vestibular system. In recent decades, a large literature has emerged concerning the role of vestibular inputs to the hippocampus (see Smith, 1997, for an early review). Aside from a few studies with humans (e.g., Vitte et al., 1996; Brandt et al., 2005), the evidence for a major role of the vestibular system in hippocampal function has come from animal studies involving vestibular stimulation or lesions (e.g., Ossenkopp and Hargreaves, 1993; Horii et al., 1994; Sharp et al., 1995; Russell et al., 2006; Tai et al., 2012). The primary labyrinthine inputs to the hippocampus and the topographical memory system in general are from the lateral/horizontal semicircular canals (Taube et al., 1996), which signal angular rotation of the head in the plane of the Earth’s surface (i.e., the domain of the topographical as opposed to gravitational orientation system). The hippocampus also receives inputs from the utricle (Cuthbert et al., 2000), which signals linear acceleration in the plane of the Earth’s surface. The importance of the vestibular inputs to the hippocampus may be due to the fact that the head is the anchor for the topographical memory system (Previc, 1998)—hence, the reference to it (“heading”) when describing movements in topographical space.

Despite the above links, there is no clear evidence that vestibular function is related to topographical memory in normal humans. The purpose of this study was to assess the relationship between various measures of vestibular and related oculomotor function and topographical memory in a sample comprised of cognitively and physically “normal” elderly participants (70–85 years). This age group was selected because of the recent hypothesis that declining vestibular function in the elderly may be associated with the onset of Alzheimer’s disease (Previc, 2013), which is heralded by a loss of topographical orientation and memory and atrophy and/or metabolic deactivation of key components of the topographical neural network (Huang et al., 2002; Johnson et al., 2005; Berti et al., 2010; Pengas et al., 2012; Lithfous et al., 2013). Because it has proven difficult to test individuals with dementia to determine the presence of vestibular loss, the present study tested ostensibly “normal” older individuals to determine any relationship between subtle topographical memory loss and vestibular function.

To test topographical memory, three tests were used: the Camden Topographical Recognition Memory Test (CTRMT), a computerized topographical mental rotation test (TMRT) similar to the “four mountains” test used by Bird et al. (2010) and Hartley and Harlow (2012), and a virtual version of the widely used Morris water maze used in animals (Sharma et al., 2010) and humans (e.g., Moffat and Resnick, 2002; Brandt et al., 2005). While the CTRMT has not been specifically linked to hippocampal function, scene memory in general has been linked to the hippocampus (Epstein and Kanwisher, 1998; Arcaro et al., 2009; Bonnici et al., 2012). Memory for scenes requiring topographical mental rotation is also dependent on the hippocampus (Hartley and Harlow, 2012), and the critical role of the hippocampus in the water maze has been repeatedly shown in both animals (Sharma et al., 2010) and humans (Brandt et al., 2005). The standard vestibular and oculomotor tests used in this study, some of which have been linked to cognitive impairment in Alzheimer’s disease (Previc, 2013), included visual pursuit (VP), optokinetic nystagmus (OKN), the actively generated vestibulo-ocular reflex (VOR), the sensory orientation test for postural control, and two measures of rotational memory—perceived rotation error in degrees and correctly perceived direction of rotation.

The relationships among the various topographical and vestibular measures were explored using a combination of correlational and multiple regression analyses.

## Method

### Participants

A total of 25 individuals between the ages of 70 and 85 (*M* = 76.37, *SD* = 4.47) participated in this study. The sample consisted of 16 females and 9 males. Nine of the 25 participants were of Hispanic origin and 21 were Caucasian, with 3 Asian-Americans and 1 designated “other”. In terms of education, there were 7 participants with high school degrees, 6 with undergraduate degrees, and 12 with graduate degrees. All participants were prescreened for dementia, with anyone scoring less than 19 on the Montreal Cognitive Assessment (MoCA) excluded from the study. The MoCA scores ranged from 19 to 28 (*M* = 24.88, *SD* = 2.42).

All participants were prescreened for any previous balance or vestibular problems, and only two had any previous vestibular problems, both involving single episodes of dizziness or vertigo many years previously (These two participants each scored within one standard deviation of the mean or above on five of six “vestibular” tests, with one scoring low on VP gain and the other scoring low on the postural test—see later descriptions). Although no tests of visual function were performed, no participant reported difficulty in seeing any of the stimuli during the various tests. Anyone with neck surgery or hip replacement surgery was excluded, although knee replacements were permitted if they were not within the past year and were not causing the person any pain. Other exclusionary criteria included a history of brain trauma (including stroke, seizures, or traumatic brain injury), a diagnosed neurological condition, or current use of psychoactive drugs. Caffeine use not exceeding three cups of coffee or its equivalent per day, moderate social consumption of alcohol, and use of nonnarcotic pain medication was acceptable for inclusion into the study.

### Testing sites and equipment

All spatial memory testing was performed in the neuropsychology office of Dr. Michael Roman in San Antonio, TX and required approximately 90 min. Two of the tests created for this study were computerized versions of previously used tests—the virtual pond maze (VPM), which tested spatial navigation and memory, and the topographical mental rotation test, which required participants to remember the original view of a scene and then rotate it mentally. Both of these tests were executed using a Hewlett-Packard Compaq NC6400 computer under moderately dim illumination with the participant seated in a quiet room. The third spatial memory test was the CTRMT, which was administered from a test booklet by the neuropsychologist sitting directly across from the participant in a well-lit room.

The “vestibular” tests were conducted at the Ear Institute of Texas clinic in San Antonio, TX and required slightly less than one hour on average to complete. The OKN, VOR, and VP tasks were all administered using the Vorteq system (Micromedical, Chatham, IL). This system contains an inertial head-tracker for measuring the motion of the head in space and an infrared camera for measuring the position of each eye in the orbit. These tests were all conducted in darkness, aside from illumination of the stimuli, and were preceded by a calibration of the system using the fixation stimulus positioned at five positions on the screen (center and 21.8° right, left, up and down, at a viewing distance of 160 cm). The Sensory Orientation Test (SOT) tested balance under various sensory conditions and made use of the Equitest system (Neurocom, Portland, OR). Finally, the rotational memory test was administered on a barber-style chair locked in place on a rotating industrial turntable whose motion was controlled electronically (Carousel, Monrovia, CA). The turntable had an accuracy of ~0.5° for the profiles and velocities and displacements used in this study (25°/s; up to 40° left or right). When seated in the chair, which was centered on the turntable, the participant was situated at the approximate radius from a large white circular Mylar half-screen (diameter = 155 cm) containing a scale that measured laser-pointing accuracy to 0.5°.

### Tests

#### Camden Topographical Recognition Memory Test (CTRMT)

This task required participants to view photographs of 30 urban scenes, presented according to the standard instructions (Warrington, 1996) in which participants viewed each scene for up to 3 s and had to determine if the photograph was shot by an amateur or a professional. Immediately after the completion of the presentation sequence, participants were shown each of the same 30 scenes in a different order along with two similar versions of it shot from different perspectives or poses. In this three-alternative self-paced forced-choice recognition task, participants were required to point to the scene that was actually presented and to avoid guessing. Each score was based on the total correct out of 30.

#### Topographical Mental Rotation Test (TMRT)

**Figure 1:**
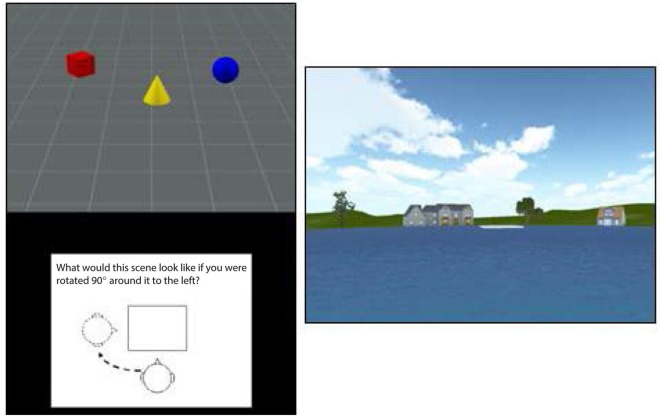
**An illustration of the stimuli used in the TMRT task (left) and pond maze (right)**. The figure at right shows the view from one of the six starting points in the pond task, with the platform shown in white in the center the pond image. The platform was visible on one-third of trials while it had to be reached from memory on the other two-thirds of trials.

Participants viewed a scene containing a set of three objects (e.g., red cylinder, blue sphere, green cube) and were then shown a figure instructing them to rotate their viewpoint 90° left, 90° right, or 180° opposite (see Figure 1, left panel). They were then shown three scenes from each of the viewpoints and asked to use the mouse to click on the image that depicted the correct viewpoint shift. A yellow box indicated their choice while a green box (only presented during practice trials) showed the correct viewpoint. Participants had 12 s to view each scene and 14 s to make their response, with a timer appearing during the last 5 s of the forced-choice interval. After being presented with instructions, which included demonstrations with actual objects, participants viewed a set of 12 practice trials (they could view a second set if they chose to) and then were presented with 15 test trials. Each score was based on the total number of correct responses out of 15.

#### Virtual Pond Maze (VPM)

In the pond maze, participants started from one of six locations around a virtual pond. Each starting location faced a different set of virtual buildings and vegetation to provide distinct spatial landmarks (see Figure 1, right panel). Participants were required to navigate using only the left and right cursor arrows to a fixed platform slightly offset from the middle of the pond, with the forward speed set by the software at a simulated 7 m/s. The location and size of the platform was set such that participants had to make at least one correction to reach the platform from each of the starting points. After approximately 20 min of instructions and practice, participants were tested on 18 trials, consisting of one platform-visible trial followed by two platform-nonvisible trials, in which spatial memory was required to reach the platform. Performance on the 6 visible and 12 nonvisible trials was measured by the time taken to reach the platform, the virtual distance traveled, and the number of corrections made. The starting position on each trial varied in a random-without-replacement order, such that all starting positions were sampled twice in the nonvisible conditions and once in the visible conditions. Because the number of corrections involved more strategy than memory and the virtual distance was almost perfectly correlated with time to reach the platform, only the latter was used in the final analysis. Also, because time taken to reach the platform on the visible trials showed little variation among participants, presumably because forward speed was set by the software, only time to reach the platform on the nonvisible trials was used to assess navigational memory.

#### Visual pursuit

In the VP task, participants were required to follow a small red square as it moved from left to right. They were instructed to not move their head, and the experimenter additionally held the head in place from behind to prevent it from moving. The small red square had a diameter of 0.5° and moved from 21.8° left to 21.8° right at increasing sinusoidal velocities (0.1–0.4 Hz). Pursuit gain relative to the movement of the square was recorded at the highest frequency (0.4 Hz) and averaged across the left and right eyes.

#### Optokinetic nystagmus

In the OKN task, participants were required to gaze at an imaginary fixation spot in the center of the screen as columns of dots swept past at a velocity of 30°/s, first leftward then rightward. Each dot was 3.96° in diameter and each column was comprised of five dots spanning 37° vertically, with an inter-column distance of 13.34°. Participants were instructed to count the number of times a dot column crossed his or her imaginary fixation spot during a 30-s interval. They were also instructed to not move their head, with the experimenter again holding the head in place from behind to help prevent it from moving. The gain of the slow phase of the OKN relative to the movement of the dots was averaged across both eyes and both directions of dot-field motion.

#### Vestibulo-ocular reflex

The VOR was measured while participants fixated on the same red square as in the VP task, which remained stationary in the center of the visual field. They actively generated left-right head movements to the sound of a metronome at frequencies beginning at 1 Hz and increasing to 3 Hz (i.e., within the natural range of head movements). Only data when the head was moving at the proper frequency were used for analysis. Because it took some delay for the participant to begin synchronizing the head autorotations to the starting frequency of 1 Hz and because many participants could not easily generate the proper head motions at 3 Hz, only the VOR gains at 2 Hz were used in the final analysis. VOR gain was averaged across two trials and both eyes. Phase of the VOR was also recorded, but it was not analyzed because the reliability was very low (see Results section).

#### Postural control

Postural tests were conducted using the Equitest’s Sensory Orientation Test (see http://resourcesonbalance.com/neurocom/products/EquiTest.aspx). The SOT provides an “equilibrium score” based on variations in the center-of-pressure under six different postural conditions in which the visual surround is either fixed or moves with the person’s postural axis (sway-referenced) or the platform is fixed or moves with the person (sway-referenced). The six conditions involve: (1) visual scene and platform both fixed; (2) eyes-closed with platform fixed; (3) sway-referenced visual scene with platform fixed; (4) visual scene fixed with sway-referenced platform; (5) eyes-closed with sway-referenced platform; (6) sway-referenced visual scene with sway-referenced platform. Equilibrium scores were recorded for two trials in each condition, usually the first and second trials. Occasionally a third trial was run if the participant was outside of normal levels or suffered a fall on the first trial, which was almost entirely restricted to conditions 5 and 6. During each condition, participants were situated in a safety harness while their feet were evenly spaced from the center point and facing forward. Condition 1 is considered the baseline condition (normal standing), whereas Condition 5 relies on vestibular inputs given that visual and somatosensory inputs (the other two major sensory inputs to the postural control system) are eliminated due to the fact that the eyes are closed and the platform moves with the participant’s postural sway (Black et al., 1995). Vestibular function was assessed in the standard manner by calculating the ratio of postural sway in Condition 5 to Condition 1.

#### Rotational memory

In this task, participants were seated in a barber chair that was locked and secured to the rotating turntable underneath. Participants were blindfolded and deprived of auditory inputs by means of ear plugs and ear muffs, which required a reliance on vestibular and, to a lesser extent, somatosensory cues. Participants received a sequence of nine trials in which they were rotated from the same starting point. Four of these trials involved single left or right rotations (to +/−30° or +/−40°), four involved combinations of eight left-right rotations (again ending at +/−30° or +/−40°), while the middle trial consisted of eight left-right rotations but ended up at the original starting position and served to disguise the pattern of the other eight end-positions. The five combination trials alternated with the single-rotation trials in a fixed order, with the control trial always being the fifth of nine. The nine trials consisted of the following sequences of rotations, in degrees: 1) 40R,20L,30R,20L,10R,40L,40R,30L,40R,20L (net = 30R); 2) 40L; 3) 20L,50R,20L,40R,30L,30R,40L,20R,30L,40R (net = 40R); 4) 30R; 5) 30L,50R,20L,10R,40L,10R,20L,30R,20L,30R (net = 0); 6) 30L; 7) 20R,50L,20R,40L, 30R,30L,40R,20L,30R,40L (net = 40L); 8) 40R; 9) 40L,20R,30L,20R,10L,40R,40L,30R, 40L,20R (net = 30L). On each trial, participants were instructed to point with arms outstretched to their perceived “straight-ahead” on the screen in front of them prior to the rotation, with the experimenter adjusting their arms to align the mark on the screen with the true straight-ahead. Immediately after the cessation of rotation, participants were instructed to aim the laser pointer to the spot on the screen where their original perceived straight-ahead intersected it, and the offset of the second mark from the first one reflected their rotational memory error, measured in degrees (RM°). After their laser pointer position was measured and recorded and their arms were relaxed, participants were then required to state whether they had ended up at or to the left or right of their original starting position (rotational direction memory (RM→). Participants were provided with some demonstrations and two practice trials to ensure they understood the task.

### Analysis

There were a total of 12 variables analyzed in this study. Six of them were independent variables related to vestibular or oculomotor function: VP, OKN, VOR, SOT, RM°, and RM→. Three additional demographic variables were included as covariates: age, education level (high school vs. college and above), and gender (male vs. female). The three topographical memory tasks (CTRMT, TMRT, and VPM) served as the dependent variables. In addition to descriptive statistics and bivariate correlations, backward stepwise multiple regressions were performed using SPSS software (IBM, Chicago, IL) for each topographical memory test, using the vestibular and demographic variables as predictors.

## Results

The means and standard deviations for the three topographical memory and six vestibular measures are shown in Table 1. The descriptive statistics showed a slightly less than perfect gain for the VP and optokinetic responses (0.83 and 0.88, respectively) and a slightly greater than unity gain (1.29) for the VOR, which involved actively generated head movements. On average, 1.6 of the direction judgments in the rotational memory task were incorrect, which is consistent with the fact that the mean rotational pointing error was 19.71° (*SD* > 7°) relative to the +/−30° and +/−40° endpoints. The most variable measures across participants were the TMRT and VPM and the SOT and RM°, where the standard deviation was a minimum of 30% of the mean. Part of the variability in the TMRT task was due to a very large difference between males (*M* = 12.44, *SD* = 2.3) and females (*M* = 7.8, *SD* = 3.51).

**Table 1:** **Means and Standard Deviations for the topographical and vestibular measures**.

**Variable**	***M***	***SD***
Camden Topographical Recognition Memory Test (correct out of 30)	24.44	4.3
Topographical Mental Rotation Test (correct out of 15)	9.52	3.74
Virtual Pond Maze (seconds)	34.2	11.0
Visual Pursuit (gain)	0.83	0.06
Optokinetic Nystagmus (gain)	0.88	0.15
Vestibular-Ocular Reflex (gain)	1.29	0.17
Sensory Orientation Test (ratio of conditions 5 to 1)	0.50	0.21
Rotational memory (° error)	19.71	7.43
Rotational memory (# correct out of 8 trials)	6.4	1.41

## Bivariate correlations

The bivariate correlation matrix involving the three topographical memory measures and six vestibular measures revealed only 2 of the 36 correlations to be statistically significant. The correlational analysis used the nonparametrc Spearman rank-order statistic, since a Shapiro-Wilk test showed significant violations of normality present for seven of the nine measures.

The highest correlations among the topographical memory measures themselves were between the TMRT and VPM scores (*ρ* = −0.34) and the CTRMT and VPM scores (*ρ* = −0.37), but both of these correlations were nonsignificant (The negative values indicate that as performance increased on the memory tasks, time taken to reach the platform in the maze task decreased).

The correlations among the “vestibular” measures were almost entirely nonsignificant, with only the measures most directly assessing horizontal semicircular canal performance (VOR, RM°, and RM→) significantly correlated with each other (*ρ* = −0.56, *p* < 0.01 for RM→/RM° and *ρ* = 0.53, *p* < 0.01 for RM°/VOR). The significant negative correlation between VOR gain and degrees of rotational memory indicates that higher VOR gains were associated with poorer rotational memory performance. The correlations among the vestibular measures and between the vestibular and topographical measures suffered partly because the reliabilities of the vestibular measures themselves were not very high. For those measures where multiple trials were gathered, the reliabilities from one trial to the next (for the VOR and SOT measures) or from the first four trials to the second set of four (for the RM° and RM→ measures) ranged from 0.26 and 0.43 for the RM→ and RM° measures, respectively, to 0.49 and 0.62 for the VOR gain and SOT measures, respectively. It is impressive, therefore, that some of the *inter-vestibular* measures attained correlation coefficients in the range of the reliability of the individual measures themselves (>0.5).

Finally, there were only two correlations out of 18 between the vestibular measures and the topographical memory measures that exceeded 0.3 (*ρ* = 36 for RM→/TMRT and *ρ* = −0.34 for RM→/VPM); both of these were nonsignificant, however.

## Multiple regressions

The regression models for the TMRT and VPM measures are listed in Tables 2A and 2B. The regression model for the CTMRT measure was excluded because, despite an *R* value of 0.575, it did not prove statistically significant. For the TMRT measure, the significant regression model with the largest number of predictor variables included RM→, gender, VP, SOT, OKN, RM°, education level, VOR, and age. The combined *R* was 0.78 (*R*^2^ = 0.608), while the largest adjusted *R*^2^ (which takes into account the number of variables and sample size) was for the combination of RM→ and gender (*R*^2^ = 0.504). The significant regression was based mainly on the fact that poorer rotational memory performance was associated with poorer TMRT scores; gender contributed as well because the TMRT means were so different between men and women. For the VPM measure, the model with the largest number of predictor variables included RM→, SOT, RM°, education level, and VOR. The combined *R* was 0.661 (*R*^2^ = 0.437), while the largest adjusted *R*^2^ was 0.289, for those same five predictors. The regression was based on the negative relationships of RM→, RM°, SOT, and educational level with the time to reach the platform and the positive relationship of VOR gain with time to reach the platform (the latter indicating that higher VOR gains were associated with poorer VPM performance).

**Table 2A:** **Regression analysis for the Topographical Mental Rotation Test**.

**Variables**	***R***	***R^2^***	***Adj. R^2^***	***df***	***F***	***P***
RM→; Gender;VP;SOT;OKN;RM°;Edu;VOR;Age	0.780	0.608	0.373	9,15	2.59	0.039
RM→; Gender;VP; SOT;OKN; RM°;Edu;Age	0.780	0.608	0.412	8,16	3.1	0.026
RM→; Gender; VP; SOT; RM°; Edu; Age	0.779	0.607	0.445	7,17	3.75	0.012
RM→; Gender; VP; SOT; RM°; Age	0.778	0.605	0.473	6,18	4.59	0.005
RM→; Gender; SOT; RM°; Age	0.762	0.580	0.469	5,19	5.25	0.003
RM→; Gender; RM°; SOT	0.754	0.568	0.481	4,20	6.52	0.002
RM→; Gender, RM°	0.730	0.532	0.465	3,21	7.97	0.001
RM→; Gender	0.710	0.504	0.459	2,22	11.17	0.000

**Table 2B:** **Regression analysis for the Virtual Pond Maze**.

**Variables**	***R***	***R^2^***	***Adj. R^2^***	***df***	***F***	***P***
RM→; RM°; VOR, SOT, Edu	0.661	0.437	0.289	5,19	2.95	0.039
RM→; RM°; VOR, SOT	0.628	0.394	0.273	4,20	3.26	0.033
RM→; RM°; VOR	0.557	0.310	0.212	3,21	3.15	0.046
RM→; RM°	0.503	0.253	0.185	2,22	3.73	0.04
RM→	0.404	0.164	0.127	1,23	4.50	0.045

## Discussion

The results of this study reveal that there is a linkage between vestibular function and topographical memory in a normal elderly human population. Because this was a correlational study, no causal inference can be made about the vestibular role in topographical memory on the basis of these results alone. However, previous evidence from animal and human clinical studies does suggest a causal contribution of vestibular function to topographical memory.

The bivariate correlation matrix revealed modest but nonsignificant relationships among the three topographical measures and much stronger relationships among the three measures most directly linked to horizontal semicircular canal function (VOR, RM°, RM→). It is somewhat surprising that the correlations among the three topographical memory measures were not stronger, given that all have been linked to hippocampal/parahippocampal processing. However, it is possible that they tap into different aspects of the topographical memory process. For instance, only the TMRT measure showed a large difference between males and females, as found in other mental rotation tasks (Nazareth et al., 2013) involving the inferior parietal lobe. Also, the Camden topographical memory test involves less active mental transformations than do the pond maze and topographical mental rotation tasks.

It was not surprising that the correlation between the two rotational memory measures was significant, in that the measure of rotational direction occurred after the participant had made his or her mark with the laser pointer to indicate the original starting direction. The high correlation of rotational direction accuracy with VOR gain was more impressive in that it was about the same as the individual reliabilities for the two measures. It is noteworthy that the correlation between VOR gain and rotational accuracy was negative, with higher gains associated with lower accuracy. Unlike the passively generated VOR, actively generated VORs by means of head autorotation tend to have above-unity gains (Hirvonen et al., 1997), and it may be speculated that higher active VOR gains provided by corollary discharge boost performance in those individuals with diminished canal function. It is unlikely that the higher gains in individuals with poorer overall vestibular function were caused by a difference in the amplitude of the head autorotations, since preliminary measurements indicated that the size of the head movement did not affect actively generated VOR gain.

The relationship between the various measures of vestibular function and the topographical memory measures was more complex. On the one hand, no vestibular measures correlated significantly with any of the topographical memory measures. On the other hand, the significant regression models incorporating the greatest number of vestibular and oculomotor predictors and adjusted for gender, age, and education accounted for over 60% of the variance for the TMRT test and over 40% of the variance for the VPM measure. As previously noted, it is likely that the regression predictions would have been even better had they not been limited by the reliability of the vestibular measures themselves. On the other hand, there is a danger in incorporating a large number of variables with a relatively small sample size of 25, although even the adjusted *R*^2^, which takes into account number of variables and sample size, proved impressive for the TMRT measure. It should be noted that the three measures of horizontal semicircular canal function tended to have slightly better predictive power in the regression analyses than did the SOT measure of vestibularly mediated postural control. This is consistent with the fact that the major vestibular inputs to the hippocampus emanate from the semicircular canals, signaling head rotation in the place of the Earth’s surface (Taube et al., 1996; Previc, 2013).

A large number of human and animal studies have linked the vestibular system and hippocampus (Ossenkopp and Hargreaves, 1993; Horii et al., 1994; Sharp et al., 1995; Vitte et al., 1996; Taube et al., 1996; Brandt et al., 2005; Russell et al., 2006; Tai et al., 2012) as well as the vestibular system and topographical memory and other functions subserved by the hippocampus (Taube et al., 1996; Vitte et al., 1996; Brandt et al., 2005). The results of this study demonstrate that vestibular function and topographic memory are clearly related, but it is not clear why. In contrast to evidence that vestibular damage induces behavioral, neuroanatomical, and neurophysiological alterations in the topographical memory system (Ossenkopp and Hargreaves, 1993; Brandt et al., 2005), it has never been shown that hippocampal or neocortical damage seriously disturbs vestibular function; hence, it is more likely that the causal direction flows from the vestibualar system. Because, however, both the vestibular system and the topographical memory system can be affected by general physiological deterioration (e.g., cardiovascular decline, diabetes, traumatic brain injury) (see Previc, 2013), it is also possible that the vestibular-topographical relationship can be explained by an outside factor affecting each separately.

The clinical implications for estimating any vestibular contribution to dementia of the Alzheimer’s type (see Previc, 2013) are also unclear. As noted in the Introduction, topographical memory impairment, whether or not caused by vestibular dysfunction, is one of the earliest signs of Alzheimer’s disease (Huang et al., 2002; Johnson et al., 2005; Berti et al., 2010; Pengas et al., 2012; Lithfous et al., 2013). By design, none of the participants in this study tested positive for dementia, but several of them showed clear impairments in topographical memory and may be at risk for developing more severe cognitive decline in the future. It cannot presently be ascertained whether a topographical memory and/or vestibular/oculomotor model could be used with diagnostic accuracy to predict who might later develop Alzheimer’s. If, however, assessment of vestibular function can at least help identify those individuals at elevated risk for developing Alzheimer’s, vestibular therapy, general exercise, and perhaps other vestibular interventions may serve as preventive measures to stave off the later cognitive decline (see Previc, 2013).

Unfortunately, it has been difficult to test Alzheimer’s patients for vestibular performance. It can be a challenge to test even many normal elderly participants because of difficulties in moving their hips, knees and neck, keeping their eyelids from obscuring the view of the eye, and a host of other problems. That makes the rotational direction measure even more potentially valuable in this regard. It was the vestibular measure best correlated with the topographic memory measures and also the easiest to administer in that it did not involve any motor response on the part of the participant. This measure should be considered as a standard part of any otolaryngological assessment in the elderly population.

In summary, this study involving normal elderly participants has confirmed previous findings from the animal and human clinical literatures that a relationship between vestibular function and hippocampally mediated topographical memory exists. Future studies are required to better understand this relationship in both normals and those at greatest risk for Alzheimer’s disease.

## Conflict of interest statement

The authors declare that the research was conducted in the absence of any commercial or financial relationships that could be construed as a potential conflict of interest.
